# Assessing volatile organic compounds exposure and chronic obstructive pulmonary diseases in US adults

**DOI:** 10.3389/fpubh.2023.1210136

**Published:** 2023-07-05

**Authors:** Jia-jie Lv, Xin-yu Li, Yu-chen Shen, Jian-xiong You, Ming-zhe Wen, Jing-bing Wang, Xi-tao Yang

**Affiliations:** ^1^Department of Interventional Therapy, Multidisciplinary Team of Vascular Anomalies, Shanghai Ninth People’s Hospital, Shanghai Jiao Tong University, Shanghai, China; ^2^Department of Vascular Surgery, Shanghai Ninth People’s Hospital, Shanghai Jiao Tong University, Shanghai, China; ^3^Department of Neurosurgery, Shanghai Ninth People’s Hospital, Shanghai Jiao Tong University, Shanghai, China

**Keywords:** volatile organic compounds (COV’s), chronic obstructive pulmonary diseases (COPD), the National Health and Nutrition Examination Survey (NHANES), US adults, cross-sectional study

## Abstract

**Background:**

Volatile organic compounds (VOCs) are a large group of chemicals widely used in People’s Daily life. There is increasing evidence of the cumulative toxicity of VOCs. However, the association between VOCs and the risk of COPD has not been reported.

**Objective:**

We comprehensively evaluated the association between VOCs and COPD.

**Methods:**

Our study included a total of 1,477 subjects from the National Health and Nutrition Examination Survey, including VOCs, COPD, and other variables in the average US population. Multiple regression models and smooth-curve fitting (penalty splines) were constructed to examine potential associations, and stratified analyses were used to identify high-risk groups.

**Results:**

We found a positive association between blood benzene and blood o-xylene concentrations and COPD risk and identified a concentration relationship between the two. That is, when the blood benzene and O-xylene concentrations reached 0.28 ng/mL and 0.08 ng/mL, respectively, the risk of COPD was the highest. In addition, we found that gender, age, and MET influence the relationship, especially in women, young people, and people with low MET.

**Significance:**

This study revealed that blood benzene and blood o-xylene were independently and positively correlated with COPD risk, suggesting that long-term exposure to benzene and O-xylene may cause pulmonary diseases, and providing a new standard of related blood VOCs concentration for the prevention of COPD.

## 1. Introduction

Volatile organic compounds (VOCs) constitute a heterogeneous group of minute organic molecules that occur in numerous natural and artificial sources, encompassing plants, fungi, animals, and industrial products (1–3). These compounds are characterized by their volatility, which enables their facile dispersion into the ambient environment at room temperature and pressure. Volatile organic compounds, being low molecular weight organic entities, are detectable in various biological fluids such as exhaled breath, blood, among others (4, 5). They arise from diverse endogenous and exogenous sources, which include cellular metabolism, gut microbiota, and environmental pollutants (4, 6). Over the past few years, there has been growing interest in the putative roles of volatile compounds in the development and progression of sundry diseases (7–9). Multiple studies have demonstrated that certain volatile compounds may serve as signaling molecules and modulate cellular processes like cell proliferation, differentiation, and apoptosis (7, 9, 10). They may also regulate the immune system, influence pathogen growth and survival, and impact host cell metabolism (11). The impact of volatile compounds on disease has been examined in various contexts, ranging from cancer and cardiovascular disease to infectious and neurodegenerative ailments. Volatile compounds have emerged as potential biomarkers for early detection and diagnosis of specific diseases, such as Alzheimer’s disease and lung cancer (12, 13). For example, VOCs could aid in the early detection of lung cancer, which is particularly crucial as the early symptoms of lung cancer are typically insidious and often manifest in advanced stages (12, 14). Some studies have evinced that early detection and prognosis of lung cancer may be feasible by identifying VOCs in the exhaled breath or blood of lung cancer patients (15). This technique offers several benefits, such as being non-invasive, straightforward, rapid, and cost-effective. Additionally, some volatile compounds have been associated with the onset of respiratory illnesses like asthma and chronic obstructive pulmonary disease (COPD), and VOCs may instigate or exacerbate COPD by interacting with biological mechanisms such as oxidative stress, inflammatory response, and apoptosis, which may inflict damage to lung tissue (16, 17).

Chronic obstructive pulmonary disease (COPD) is a frequent, progressive lung ailment that is characterized by sustained airflow limitation and chronic inflammation of the airways. It is among the leading causes of morbidity and mortality globally, with an estimated 251 million cases and 3.2 million deaths in 2019 (18, 19). COPD is a multifactorial, intricate disease that is intricately associated with smoking and exposure to air pollution. Other risk factors include occupational exposure to dust and chemicals, genetic predisposition, and respiratory infections (20). Despite significant advancements in our knowledge of the disease, the pathogenesis of COPD remains incompletely understood, and there is currently no definitive cure for the ailment. Recent investigations have shown that the blood volatile organic compound (VOC) profile differs between COPD patients and healthy subjects, implying that blood VOCs may have potential as a diagnostic tool for COPD (21, 22). Additionally, numerous studies have scrutinized the correlation between specific VOCs and COPD severity, exacerbation risk, and mortality (23). Despite burgeoning evidence of the links between volatile compounds and disease, the fundamental mechanisms by which these compounds operate are still not fully comprehended. Moreover, the vast heterogeneity of volatile compounds and their origins poses a formidable challenge in identifying and ascertaining their impact on human health. Therefore, it is crucial to devise relevant environmental health guidelines predicated on the association between blood VOC biomarkers and diseases, in order to accomplish early disease prevention and diagnosis (24, 25).

To our knowledge, no studies have been reported on the dose-risk association of volatile organic compounds (VOCs) concentration in blood with the incidence of chronic obstructive pulmonary disease (COPD), and the specific dose-risk relationship between the two requires further exploration. We postulate that blood VOCs could elicit pulmonary pathological changes that impact COPD risk in the US population. In this study, we conducted a secondary analysis of data from the National Health and Nutrition Examination Survey (NHANES), with multiple confounding factors being controlled for. Our objective was to examine the relationship between blood VOCs and COPD risk in the US population and to establish a novel standard for relevant blood VOCs concentration to prevent COPD.

## 2. Materials and methods

### 2.1. Data source and study population

**Figure 1:**
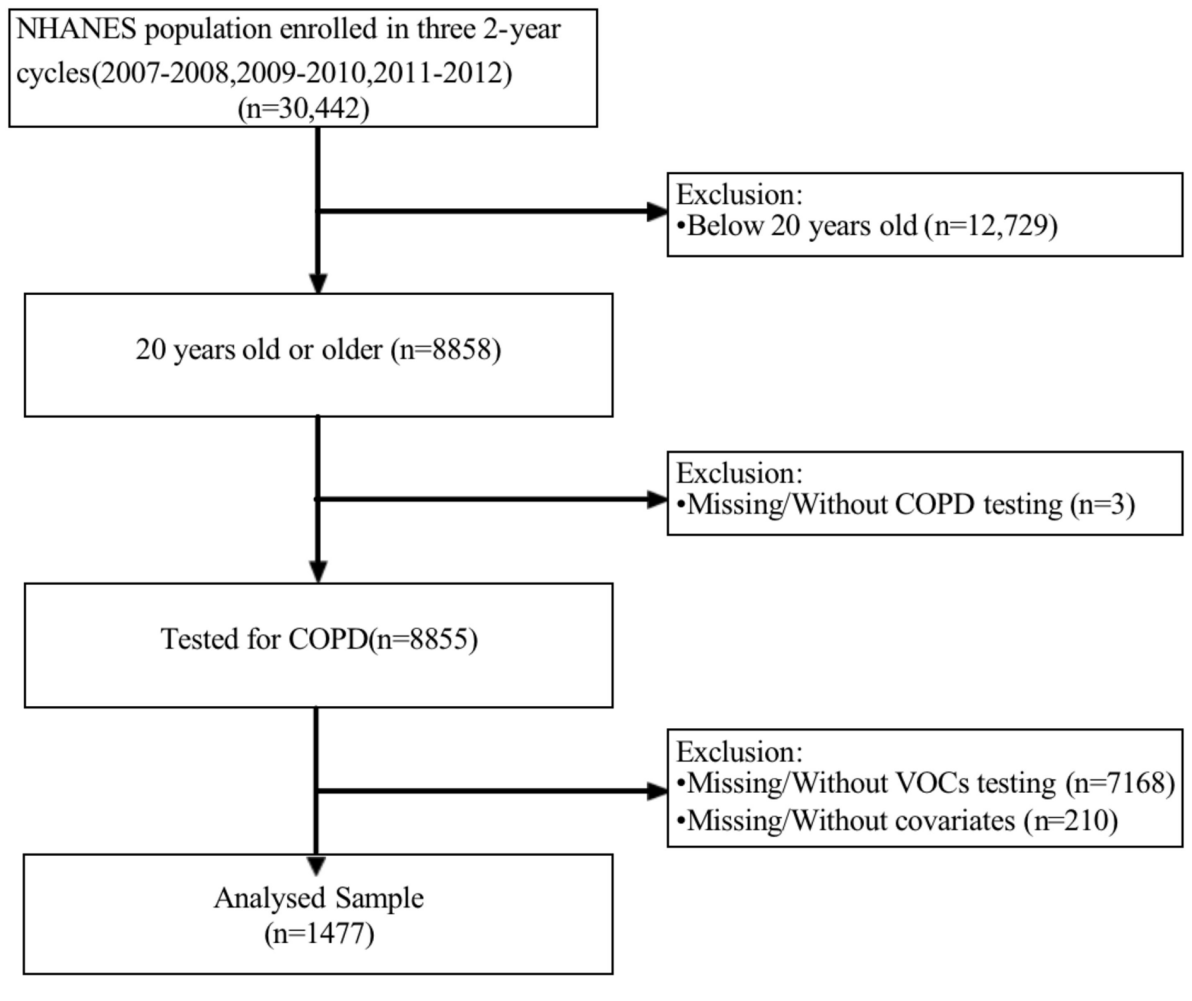
Flowchart for selecting analyzed participants.

The National Health and Nutrition Examination Survey (NHANES) is a comprehensive, interdisciplinary survey program initiated by the Centers for Disease Control and Prevention (CDC) to evaluate the health and nutrition status of U.S. residents. The overarching objective of NHANES is to gather, scrutinize, and publish data on the health, nutrition, and environmental exposures of U.S. residents. NHANES has been administered annually since the 1960s and encompasses individuals of all ages across the United States. For the present analysis, we amalgamated three survey periods (i.e., 2007–2008, 2009–2010, and 2011–2012) to generate estimates with heightened precision and less sampling error. We confined our study sample to non-pregnant participants aged between 20–80 years (*N* = 30,442). Subsequently, we excluded participants without complete information on COPD (*N* = 3) and blood VOCs (*N* = 7,168). Eventually, a total of 1,477 eligible individuals from the National Health Service were incorporated in our analysis (Figure 1). The NHANES Research Ethics Review Committee granted approval for the NHANES research protocols for 2007–2010 (2005–2006) and 2011–2016 (2011–2017), with all participants providing written informed consent (26).

### 2.2. Assessment of VOCs

Volatile organic compounds (VOCs) are an extensive group of chemicals employed as solvents, degreasers, and cleansers in both industrial and consumer products. Measurement of VOCs encompasses the quantification of human blood VOCs and household tap water VOCs. Tap water VOCs include THMs (chloroform, bromodichloromethane, dibromochloromethane, and bromoform) and MTBE. Headspace solid phase microextraction (SPME) was implemented in combination with capillary gas chromatography and mass spectrometry to determine trace amounts of THMs and MTBE in human blood. Quantitative analysis of blood disinfection byproducts (DBPs) (chloroform, bromodichloromethane, dibromochloromethane, and bromoform) and MTBE was accomplished by combining capillary gas chromatography (GC) and high-resolution mass spectrometry (MS) techniques with ion mass detection and isotope dilution methods. VOCs (tetrachloroethylene, benzene, 1,4-dichlorobenzene, ethylbenzene, o-xylene, styrene, trichloroethylene, toluene, m-p-xylene) present in human blood were quantified using SPME in conjunction with gas chromatography and Benchtop quadrupole mass spectrometry.

### 2.3. Assessment of COPD

In NHANES, the diagnosis of COPD was made by administering a spirometry test to participants. Spirometry is a lung function test that gauges the amount and velocity of air that an individual can forcefully exhale. The NHANES spirometry protocol conforms to the American Thoracic Society (ATS) and European Respiratory Society (ERS) guidelines for spirometry. Participants with a forced expiratory volume in 1 second (FEV1)/forced vital capacity (FVC) ratio less than 0.70 post-bronchodilator use were classified as having COPD. Post-bronchodilator values are employed because a post-bronchodilator spirometer is recommended for the diagnosis of COPD. In NHANES, the vital capacity tests of participants are executed by proficient and authorized technicians (27). The data is then evaluated and interpreted by certified pulmonologists. All lung function tests conducted in NHANES follow ATS/ERS guidelines to ensure that results are standardized and precise. Once a COPD diagnosis is established, this information is recorded in the NHANES database, along with other health information amassed during the survey.

### 2.4. Covariates

The underlying covariates were determined *a priori*, based on a comprehensive literature review (28, 29). The multivariate adjustment model summarized the following confounding factors: continuous variables encompassed age, poverty income ratio, body mass index, metabolic equivalent, and comorbidity index. Categorical variables included sex (male/female), marital status (married or cohabiting/single), race/ethnicity, education, and smoking status. Comorbidities such as diabetes, congestive heart failure, coronary artery disease, chronic obstructive pulmonary disease (chronic bronchitis and/or emphysema), hypertension, and cancer were also incorporated. Each disease was scored on a scale ranging from 1 to 6, with higher scores reflecting a greater impact on a patient’s health status and prognosis. The comorbidity index score could be calculated by summing up the scores for each disease, and higher CCI scores indicated more severe cases of multiple diseases.

### 2.5. Statistical analysis

According to the CDC complex NHANES data analysis guide,^1^ each NHANES participant was assigned a sample weight. Therefore, to account for masking variance, we used the recommended weighting method in our analysis. Data are presented as mean ± SD or proportions. To compare differences between the COPD and non-COPD groups, we utilized a weighted t-test for continuous variables and a weighted χ2 test for categorical variables. Our statistical analysis employed three main strategies to examine whether blood VOCs were associated with COPD. Initially, we utilized simple and easily solvable weighted univariate models, followed by multiple logistic regression models. The Crude Model and Model 1 (adjusted for sex and age only) were estimated. In the final model (Model 2), we further adjusted for BMI, PIR, level of education, marital status, alcohol intake, metabolic equivalent, comorbidity index, and smoking status. We observed statistical differences between blood benzene, xylene, and COPD. To illustrate the nonlinear relationship between blood benzene, xylene, and COPD, we performed a smooth curve fitting (penalty spline method) to explore the potential nonlinear relationship between blood benzene, xylene, and COPD. Furthermore, we investigated the extent of differences between groups based on sex, age, and metabolic equivalent. Ultimately, we constructed subgroup analyses, represented by blood benzene, to determine the stratified association between blood benzene and COPD using hierarchical multiple logistic regression.

All analyses were performed using the statistical software packages R (Version 4.2.3) and SPSS. *p* < 0.05 was considered to be statistically significant.

## 3. Results

### 3.1. Basic characteristics of participants

Table 1 depicts the baseline characteristics of the selected population in the NHANES dataset (2007–2012), which was distributed with weighting. The variables comprised sociodemographic variables, laboratory data, medical examination and personal life history, blood VOCs, and COPD data. The study population was bifurcated into the COPD group and the non-COPD group based on the presence or absence of COPD. Marital status, poverty/income ratio, BMI, metabolic equivalent of task (MET), comorbidity index, and smoking status exhibited no significant differences between the two groups (*p* > 0.05). However, there were significant differences in race/ethnicity, education level, and alcohol intake between the two groups (*p* < 0.05). The population with COPD had a higher average age, which aligns with prior literature. Compared to the non-COPD group, the distribution of VOCs, such as blood benzene, blood chloroform, blood 1, 4-dichlorobenzene, blood ethylbenzene, blood o-xylene, blood trichloroethylene, blood toluene, and blood mixed xylene, was statistically significant, which may suggest a difference in exposure between the two groups. However, there was no significant difference in exposure to VOCs such as blood tetrachloroethylene, blood bromoform, blood bromodichloromethane, blood dibromochloromethane, blood methyl tert-butyl ether, and blood styrene. Non-Hispanic whites constituted the primary population in our study.

**Table 1:** Characteristics of participants by COPD, NHANES 2007–2012.

Variable	Total	Non-COPD	COPD	P
Age	45.86	45.03	59.42	
Gender(%)				**0.97**
Female	757 (51.25)	716 (52.01)	41 (51.76)	
Male	720 (48.75)	676 (47.99)	44 (48.24)	
Race/ethnicity (%)				**0.01**
Mexican American	334 (22.61)	330 (12.08)	4 (1.37)	
Non-Hispanic Black	334 (22.61)	320 (13.81)	14 (8.44)	
Non-Hispanic White	603 (40.83)	540 (62.38)	63 (89.10)	
Other Hispanic	145 (9.82)	141 (5.24)	4 (1.09)	
Other race/ethnicity	61 (4.13)	61 (6.49)	0 (0.00)	
Marital status (%)				0.07
Living with partner	108 (7.31)	105 (6.64)	3 (5.00)	
Married	1,069 (72.38)	997 (71.79)	72 (85.18)	
Single	300 (20.31)	290 (21.57)	10 (9.81)	
Education level (%)				**0.03**
High school	583 (39.55)	545 (35.23)	38 (44.58)	
Less than high school	186 (12.62)	174 (5.94)	12 (9.88)	
More than high school	705 (47.83)	670 (58.83)	35 (45.54)	
PIR (SD)	3.11 (0.09)	3.11 (0.09)	3.10 (0.23)	0.98
BMI (SD)	28.62 (0.23)	28.67 (0.24)	27.77 (0.46)	0.1
MET (SD)	4887.47 (300.40)	4933.47 (317.83)	4040.03 (894.78)	0.36
CCI	0.81	0.74	1.84	
Alcohol intake (%)				**0.02**
Former	287 (19.43)	259 (15.17)	28 (29.25)	
Heavy	322 (21.8)	307 (22.03)	15 (20.37)	
Mild	431 (29.18)	405 (33.16)	26 (36.15)	
Moderate	218 (14.76)	207 (17.46)	11 (9.72)	
Never	219 (14.83)	214 (12.19)	5 (4.52)	
Smoking status (%)				
Former	350(23.7)	316 (20.95)	34 (38.54)	
Never	798 (54.03)	785 (58.49)	13 (14.97)	
Now	329 (22.27)	291 (20.56)	38 (46.49)	
Blood VOCS (SD)				
Blood Tetrachloroethene (ng/mL)	0.05 (0.00)	0.05 (0.00)	0.04 (0.01)	0.4
Blood Bromoform (pg/mL)	1.61 (0.31)	1.63 (0.33)	1.40 (0.32)	0.6
Blood Bromodichloromethane (pg/mL)	2.84 (0.30)	2.87 (0.31)	2.34 (0.39)	0.14
Blood Benzene (ng/mL)	0.07 (0.01)	0.06 (0.01)	0.14 (0.02)	**0.003**
Blood Chloroform (pg/mL)	16.93 (2.97)	17.32 (3.12)	10.42 (1.54)	**0.02**
Blood Dibromochloromethane (pg/mL)	1.91 (0.23)	1.93 (0.24)	1.64 (0.41)	0.49
Blood 1,4-Dichlorobenzene (ng/mL)	1.00 (0.19)	1.03 (0.20)	0.36 (0.22)	**0.05**
Blood Ethylbenzene (ng/mL)	0.04 (0.00)	0.04 (0.00)	0.07 (0.01)	**0.01**
Blood MTBE (pg/mL)	2.91 (0.63)	2.83 (0.64)	4.10 (1.68)	0.46
Blood O-xylene (ng/mL)	0.04 (0.00)	0.04 (0.00)	0.05 (0.01)	**0.03**
Blood Styrene (ng/mL)	0.11 (0.06)	0.11 (0.07)	0.08 (0.01)	0.61
Blood Trichloroethene (ng/mL)	0.01 (0.00)	0.01 (0.00)	0.01 (0.00)	**0.05**
Blood Toluene (ng/mL)	0.22 (0.02)	0.21 (0.02)	0.37 (0.05)	**0.01**
Blood m−/p-Xylene (ng/mL)	0.13 (0.01)	0.13 (0.01)	0.21 (0.03)	**0.01**

a. Values are weighted mean (SD) for continuous variables and weighted percentage for categorical variables; *p* values were derived from Student’s t test for continuous variables and the Chi-square test for categorical variables. Bold values indicate that the *P* value is less than 0.05.

b. COPD, chronic obstructive pulmonary disease; PIR, Poverty to income ratio; BMI, Body mass index; MET, metabolic equivalent; CCI, co-morbidity index; VOCS, volatile organic compounds.

### 3.2. The regression analysis between VOCs and COPD concentrations

Table 2 presents the results of the multiple logistic regression analyses for the association between blood VOCs and COPD prevalence. The analysis was adjusted for potential confounding factors, including age, sex, poverty income ratio, education level, marital status, BMI, alcohol intake, metabolic equivalent, comorbidity index, and smoking status. We found that only blood benzene and blood o-xylene were positively and statistically significantly associated with COPD prevalence. In the crude model, as blood benzene (ng/ml) and blood o-xylene (ng/ml) concentrations increased, the risk of COPD increased by 15.43 times [OR = 15.43, 95%CI (3.05, 78.10)] and 2.99 times [OR = 2.99, 95%CI (1.11, 8.06)], respectively. After adjusting for age and sex (model 1), the association remained significant, with an increased risk of COPD by 17.20 times [OR = 17.20, 95%CI (3.38, 87.47)] for blood benzene and 3.49 times [OR = 3.49, 95%CI (1.30, 9.41)] for blood o-xylene. Further adjustment for other confounding factors (model 2) increased the risk estimates for both blood benzene and blood o-xylenes. In the fully adjusted model (model 2), as blood benzene (ng/ml) and blood o-xylene (ng/ml) concentrations increased, the risk of COPD increased by 78.31 times [OR = 78.31, 95%CI (8.11, 755.81)] and 2.84 times [OR = 2.84, 95%CI (1.00, 8.08)], respectively. These findings suggest that long-term exposure to benzene and xylenes in the environment may be an independent risk factor for respiratory diseases, especially lung damage.

**Table 2:** Association of blood VOCS with COPD.

Exposure	Crude Model			Model 1			Model 2		
	OR	95% CI	*p*	OR	95% CI	*p*	OR	95% CI	*p*
Blood Tetrachloroethene	0.29	0.00, 157.39	0.69	0.17	0.00, 112.08	0.58	0.36	0.00, 127.19	0.71
*p* for trend	0.69			0.58			0.71		
Blood Bromoform	1.00	0.96, 1.03	0.78	0.99	0.94, 1.05	0.82	1.00	0.97, 1.02	0.66
*p* for trend	0.78			0.82			0.66		
Blood Bromodichloromethane	0.95	0.87, 1.04	0.25	0.96	0.88, 1.05	0.32	0.98	0.90, 1.07	0.63
*p* for trend	0.25			0.32			0.63		
Blood Benzene	15.43	3.05, 78.10	0.002	26.41	4.18, 167.00	0.002	78.31	8.11, 755.81	0.002
*p* for trend	0.002			0.002			0.002		
Blood Chloroform	0.99	0.97, 1.01	0.20	0.99	0.97, 1.01	0.24	0.99	0.97, 1.01	0.51
*p* for trend	0.20			0.24			0.51		
Blood Dibromochloromethane	0.96	0.83, 1.11	0.57	0.96	0.83, 1.11	0.58	0.98	0.85, 1.14	0.79
*p* for trend	0.57			0.58			0.79		
Blood 1,4-Dichlorobenzene	0.92	0.75, 1.13	0.39	0.93	0.78, 1.12	0.44	0.69	0.47, 1.02	0.06
*p* for trend	0.39			0.44			0.06		
Blood Ethylbenzene	4.01	0.74, 21.71	0.10	2.64	0.08, 91.51	0.57	5.91	1.17, 29.71	0.03
*p* for trend	0.10			0.57			0.03		
Blood MTBE	1.00	1.00, 1.01	0.43	1.00	0.95, 1.05	0.91	1.01	1.00, 1.01	0.07
*p* for trend	0.43			0.91			0.07		
Blood o-Xylene	2.99	1.11, 8.06	0.03	4.15	1.42, 12.14	0.01	2.84	1.00, 8.08	0.05
*p* for trend	0.03			0.01			0.05		
Blood Styrene	1.00	0.98, 1.01	0.47	1.03	1.00, 1.05	0.05	1.01	1.00, 1.03	0.08
*p* for trend	0.47			0.05			0.08		
Blood Toluene	1.17	0.50, 2.70	0.71	0.84	0.23, 3.15	0.79	1.20	0.76, 1.89	0.40
*p* for trend	0.71			0.79			0.40		
Blood m−/p-Xylene	1.55	0.98, 2.45	0.06	0.55	0.04, 7.47	0.64	1.74	1.10, 2.76	0.02
*p* for trend	0.06			0.64			0.02		

a. Crude Model: no covariates were adjusted.

b. Model 1: adjusted for age; gender.

c. Model 2: adjusted for age; gender; race; Education level; Marital status; PIR; BMI; CCI; smoking status; alcohol intake.

### 3.3. Nonlinearity analysis using RCS

**Figure 2:**
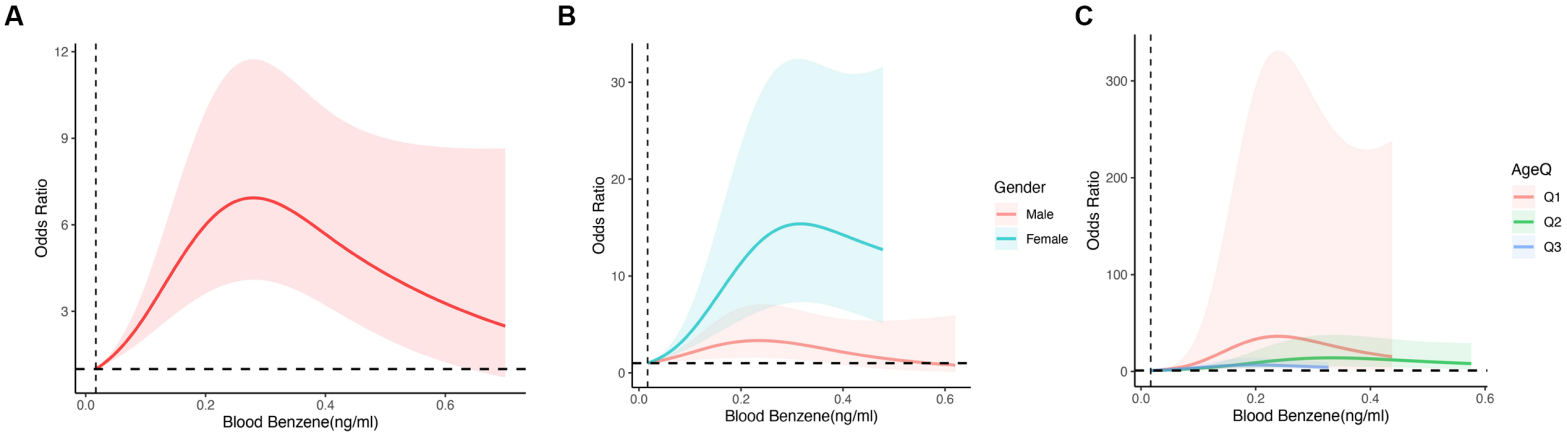
The odds ratio of COPD with Blood Benzene, NHANES 2007–2012. OR: Odds ratio; CI: Confidence interval. **(A)** The non-linear relationship between Blood Benzene and COPD. **(B)** The odds ratio of COPD with Benzene by Gender. **(C)** The odds ratio of COPD with Benzene by Age; Age Q: Q1:20,39; Q2:39,59; Q3:59,80.Adjusted for age, gender, race, educational level, marital status, PIR, BMI, smoking status, alcohol intake, diabetes and hypertension. The shaded part represents the 95% confidence interval.

**Figure 3:**
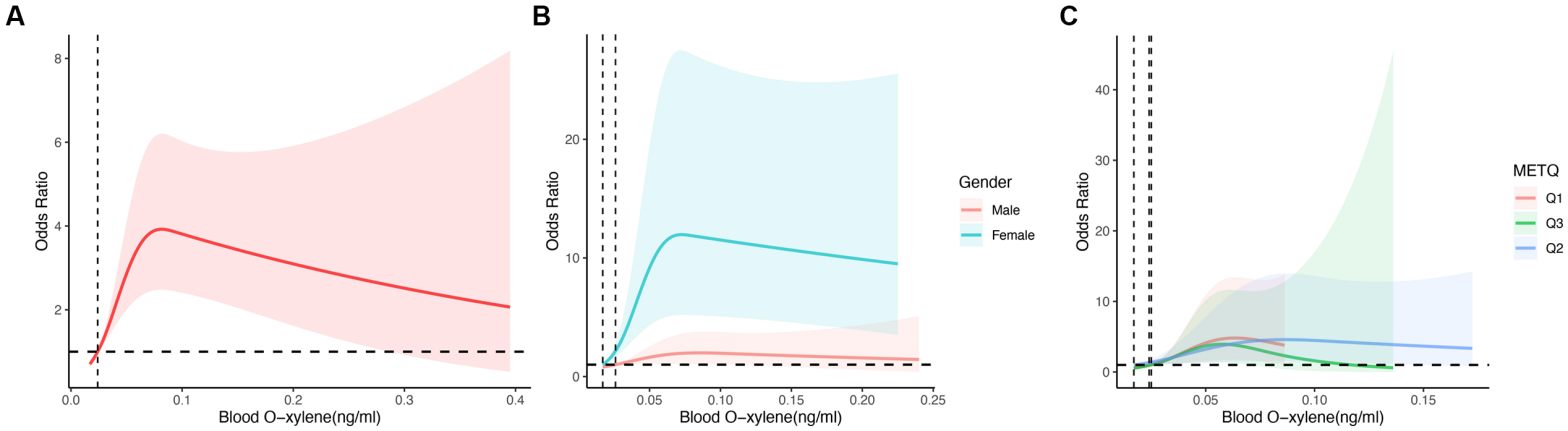
The odds ratio of COPD with Blood O-xylene, NHANES 2007–2012. OR: Odds ratio; CI: Confidence interval. **(A)** The non-linear relationship between Blood O-xylene and COPD. **(B)** The odds ratio of COPD with O-xylene by Gender. **(C)** The odds ratio of COPD with O-xylene by MET; METQ: Q1:40,600; Q2:600,3,000; Q3:3000,46,200. Adjusted for age, gender, race, educational level, marital status, PIR, BMI, smoking status, alcohol take, diabetes and hypertension. The shaded part represents the 95% confidence interval.

To examine the correlation between blood benzene and blood o-xylene concentrations and chronic obstructive pulmonary disease (COPD), we utilized smooth curve fitting, employing the penalty spline method to analyze the nonlinear relationship between blood benzene and blood o-xylene concentrations and COPD (Figures 2, 3), respectively. Our analysis using a constrained spline model demonstrated a positive association between blood benzene and blood o-xylene concentrations and the incidence of COPD, even after adjusting for confounding factors such as age, sex, race, education, marriage, family size, family income, body mass index, smoking, drinking, and comorbidity index. Specifically, when the blood benzene concentration was less than 0.28 ng/mL, the risk of COPD increased with an increase in blood benzene concentration. The highest risk of COPD was observed when the blood benzene concentration reached 0.28 ng/mL. However, when the blood benzene concentration was greater than 0.28 ng/mL, the risk of COPD decreased with an increase in blood benzene concentration. Nonetheless, overall trends indicated a positive association between blood benzene concentrations and COPD (Figure 2A). Furthermore, we observed a significant correlation between the blood benzene concentration and the incidence of COPD by sex and age group (Figures 2B,C). Specifically, this positive relationship was more prominent among women and younger individuals, suggesting that the association between blood benzene concentration and the incidence of COPD may be affected by gender and age.

Similarly, we observed an increased risk of COPD with an increase in blood o-xylene concentration less than 0.08 ng/mL. The highest risk of COPD was observed when the blood o-xylene concentration reached 0.08 ng/mL. However, when the blood o-xylene concentration was greater than 0.08 ng/mL, the risk of COPD decreased with an increase in the blood o-xylene concentration. Nonetheless, overall trends indicated a positive correlation between blood o-xylene concentrations and COPD (Figure 3A). Moreover, we identified a significant correlation between blood o-xylene concentration and the incidence of COPD in different sex and metabolic equivalent of task (MET) groups (Figures 3B,C). Specifically, this positive relationship was more prominent among women and those in the low MET group, suggesting that the association between blood o-xylene concentration and the incidence of COPD may be influenced by gender and MET.

### 3.4. Stratified associations between COPD and blood benzene

**Figure 4:**
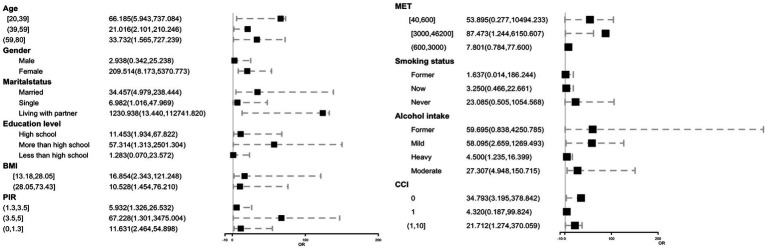
Forest plot of COPD across blood benzene with subgroup, NHANES 2007–2012. OR, Odds ratio; CI, Confidence interval.

We also conducted further analyses to investigate the stratified associations between COPD and blood benzene concentrations in specific subgroups, including age, sex, marital status, education, body mass index (BMI), poverty-income ratio (PIR), metabolic equivalent of task (MET), alcohol intake, and comorbidities (Figure 4). Surprisingly, we found a significant association between blood benzene concentration and COPD in specific subgroups. Specifically, blood benzene concentrations were associated with a higher risk of COPD in women aged 20 to 39 years (*p* < 0.05), which is consistent with our findings from the non-linear analysis. Additionally, this positive association was also observed in individuals living with a partner, those with more education than high school, those with a high income, and those with a BMI ranging from 13.18 to 28.

## 4. Discussion

In this extensive population-based study of adults in the United States, we investigated the potential association between volatile organic compounds (VOCs) and the risk of COPD based on blood VOCs concentrations. Our findings reveal three important insights. Firstly, our analysis indicates a noteworthy association between blood VOCs concentrations and the risk of COPD, even after accounting for various potential confounders. Specifically, blood benzene and blood o-xylene were positively associated with the risk of COPD within a certain range of blood levels. Secondly, our results showed that gender, age, and metabolic equivalent of task (METS) could influence the association, particularly in females, younger individuals, and those with low METS. Finally, we found that the risk of COPD was highest when the blood benzene and blood o-xylene concentrations reached 0.28 ng/mL and 0.08 ng/mL, respectively, providing a new relevant blood VOCs concentration standard for the prevention of COPD.

To date, no large-scale, systematic investigations have been conducted to examine the association between blood VOCs concentrations and chronic respiratory diseases (CRDs), particularly the concentration-risk relationship between VOCs and chronic obstructive pulmonary disease (COPD) (25, 30, 31). In the present study, our findings demonstrate a positive correlation between blood concentrations of benzene and o-xylene and the risk of COPD. Furthermore, restricted cubic spline (RCS) analysis revealed an overall positive association between VOCs and COPD, with the highest risk observed when blood concentrations of benzene and o-xylene reached 0.28 ng/mL and 0.08 ng/mL, respectively. Several factors may contribute to these results. First, as organic compounds accumulate within the body, they are metabolized and compensated for, ultimately reaching an “adaptive” state. A Chinese study (26) reported increased platelet counts in benzene-exposed subjects compared to controls. Additionally, Ahmadi et al. (27) found that red blood cell (RBC) and hemoglobin (Hb) values, as well as hematocrit (HCT) percentages, were elevated in gas station workers compared to healthy controls, suggesting that the body compensates in response to VOCs exposure. Second, the relationship between VOCs exposure and respiratory disease severity remains a topic of debate. Smargiassi et al. (28) conducted a panel study of 72 asthmatic children living near two oil refineries in Montreal, Quebec, and found no evidence of an association between lung function and exposure to ambient air pollutants, including benzene. Moreover, Chen et al. (29) performed a cross-sectional study among older adult individuals (50–71 years old) in Nanjing to compare blood levels of benzene, m-toluene/p-xylene, and o-xylene (BTEX) and other hematological parameters across different populations. Their results indicated that the highest BTEX blood levels were observed in the contaminated group, along with reductions in neutrophil, platelet (PLT), and RBC counts, HCT, mean corpuscular hemoglobin concentration (MCHC) levels, and Hb concentration compared to the control group. Lastly, metabolic enzymes encoded by corresponding genes play a crucial role in the *in vivo* metabolism of benzene and other organic compounds. The activities of these genes and metabolic enzymes are closely related to blood VOCs concentrations. A Brazilian study (32) assessed S-phenylmercapturic acid (S-PMA) and genetic polymorphisms for CYP2E1 1293G > C and NQO1 609C > T in 190 adults living within a 1 km radius of a refinery for more than three months. CYP2E1 and NQO1 are key enzymes involved in benzene metabolism. CYP2E1, a phase I enzyme, functions primarily in the liver, where it converts benzene and other benzene-derived metabolites into toxic compounds. Consequently, higher CYP2E1 activity results in increased production of benzene-derived toxins. NQO1, a phase II enzyme, primarily operates in bone marrow, reducing benzoquinones (BQs) to less toxic metabolites (33, 34). Thus, higher enzyme activity leads to decreased toxicity from benzene metabolites. The aforementioned studies demonstrated a statistically significant association between the presence of variant alleles in the NQO1 genotype and observed hematological abnormalities in exposed adults compared to controls. Based on these studies and analyses, we hypothesize that the observed reduction in COPD risk may be attributable to the compensatory or metabolic pathways activated when these compounds reach certain concentrations within the body. In conclusion, while the precise mechanism linking blood benzene and o-xylene concentrations to COPD risk remains to be elucidated, potential factors such as metabolic pathway involvement and antioxidant activity warrant further investigation to validate these hypotheses (35, 36). Furthermore, elevated levels of education and income may be associated with residing in urban areas with high pollution levels, concurrently experiencing increased stress in living and working conditions. A multitude of factors could potentially contribute to adverse health outcomes and heightened disease risk.

As mentioned above, VOCs are ubiquitous chemicals in life, and their emissions come from a wide range of sources (37–40). An increasing body of evidence suggests that VOCs may pose a risk to public health. The VOCs in this study included disinfection byproducts (chloroform, bromodichloromethane, dibromochloromethane, and bromoform), methyl tert-butyl ether (MTBE), and other VOCs (tetrachloroethylene, benzene, 1,4-dichlorobenzene, ethylbenzene, o-xylene, styrene, trichloroethylene, toluene, m-p-xylene). Disinfection byproducts are formed when chlorine reacts with organic substances in water, and are commonly found in drinking and recreational water bodies (41). They exhibit cytotoxicity, mutagenicity, teratogenicity, and carcinogenicity. MTBE was once widely used as a gasoline additive but has since been banned due to groundwater contamination. Furthermore, benzene, tetrachloroethylene, 1,4-dichlorobenzene, ethylbenzene, o-xylene, styrene, trichloroethylene, toluene, and m-p-xylene are frequently used in industry and chemical synthesis. Benzene, for example, is used in the production of DDT, phenol, and nitrobenzene, while 1,4-dichlorobenzene is used as an insect repellent and deodorant (42). Studies have also shown that occupational exposure to VOCs may be associated with cancer (43–45). While some studies have revealed that the concentration and type of VOCs may be related to the severity of COPD, and the severity of COPD can be assessed by detecting VOCs in the exhaled gas of COPD patients (23–25), the dose-risk relationship between blood VOCs concentration and COPD has not been previously reported.

Benzene and xylene are common volatile organic compounds and pollutants, and recent studies have suggested that long-term exposure to harmful substances like benzene and xylene may contribute to the development of chronic obstructive pulmonary disease (COPD). Some studies have shown an association between exposure to harmful substances such as benzene, particularly in the workplace and environment, and an increased risk of COPD. For example, Linet et al. (30, 46) explored the relationship between occupational benzene exposure and non-malignant respiratory diseases (including COPD) among Chinese workers, and the results showed that workers exposed to benzene had a 3.3 times higher risk of developing non-malignant respiratory diseases than unexposed workers, with the risk of COPD being 2.8 times higher than that of unexposed workers. This study provides evidence of a correlation between benzene exposure and the onset of COPD. Additionally, Grahn et al. (47, 48) conducted a systematic review exploring the relationship between air pollution and stroke and cardiovascular disease, and found that long-term exposure to volatile organic compounds like benzene and xylene may increase the risk of COPD. Exposure to benzene and xylene can lead to biological processes such as oxidative stress and inflammatory responses that damage lung tissue and induce or aggravate COPD. Moreover, a study conducted in Athens, Greece (31, 49), investigating the relationship between air pollution and chronic lung disease, showed that people exposed to volatile organic compounds like xylene had a higher risk of COPD than those not exposed. Overall, the relationship between blood benzene and blood o-xylene concentrations and COPD risk requires further exploration in prospective cohort studies and validation *in vivo* and *in vitro*.

The main strength of our study lies in the use of a carefully documented cohort that includes expertise in environmental science, nutrition, and clinical medicine to investigate the association between long-term exposure to VOCs and the risk of COPD based on lung damage induced by blood VOCs concentrations. Furthermore, the data were weighted to ensure that the findings were representative of the broader US population. Owing to the constraints of the database employed and the research variables chosen by the investigating institution, the most comprehensive data available for our study spans from 2007 to 2012. To derive the most robust and exhaustive conclusions, we amalgamated three survey intervals, specifically 2007–2008, 2009–2010, and 2011–2012. However, our study has some limitations that must be acknowledged. Firstly, the cross-sectional nature of the data used in our study means that the causal relationship between VOCs and the risk of COPD remains uncertain, despite some medical plausibility. Longitudinal studies or clinical trials should be conducted to confirm these associations. Secondly, although we identified high-risk groups and revealed the relationship between blood VOCs and the risk of COPD, we could not accurately determine the specific source of blood VOCs, which was also limited by population information recorded by staff. Additionally, although we adjusted for several potential confounders, the influence of other factors cannot be completely ruled out. Further large-scale, multi-center clinical studies are required to validate the correlation between blood VOCs concentrations and the risk of COPD, and to explore its mechanism and clinical applicability.

## 5. Conclusion

Our study provides a comprehensive evaluation of the association between VOCs exposure and the incidence of COPD. We discovered that blood benzene and blood o-xylene were independently and positively associated with COPD, and established the concentration relationship between blood benzene and blood o-xylene concentrations and the risk of COPD, suggesting that long-term exposure to environmental blood benzene and blood o-xylene may lead to respiratory diseases, particularly lung damage. Furthermore, our findings revealed that gender, age, and METS could influence the association, particularly in females, younger adults, and those with lower MET. Finally, our study provides new and relevant blood VOCs concentration standards for the prevention of COPD.

### Impact statement

The impact of Volatile Organic Compounds (VOCs) exposure on the development of chronic obstructive pulmonary disease (COPD) has not been widely studied. Our study provides important insights into the association between VOCs exposure and COPD incidence. We found that blood benzene and blood o-xylene concentrations were independently and positively associated with COPD, highlighting the potential health hazards of long-term exposure to these chemicals. Our study also reveals that gender, age, and metabolic equivalent of task (MET) could influence the association, particularly in females, younger adults, and those with lower MET. Our findings suggest that policymakers and public health officials should consider implementing strategies to reduce VOCs exposure, particularly in high-risk populations. Finally, our study provides new and relevant blood VOCs concentration standards for the prevention of COPD, which could help inform public health policy and guide clinical decision-making. Overall, our study provides important contributions to the literature on environmental risk factors for COPD and could have significant implications for the prevention and management of respiratory diseases in the general population.

## Data availability statement

The datasets used in this study are publicly available via https://www.cdc.gov/nchs/nhanes/index.htm (accessed on 1 April 2023).

## Ethics statement

The studies involving human participants were reviewed and approved by The NHANES Research Ethics Review Committee granted approval for the NHANES research protocols for 2007–2010 (2005–2006) and 2011–2016 (2011–2017), with all participants providing written informed consent. The patients/participants provided their written informed consent to participate in this study.

## Author contributions

All authors made substantial contributions to conception and design, acquisition of data, or analysis and interpretation of data; took part in drafting the article or revising it critically for important intellectual content; agreed to submit to the current journal; gave final approval of the version to be published; and agree to be accountable for all aspects of the work.

## Conflict of interest

The authors declare that the research was conducted in the absence of any commercial or financial relationships that could be construed as a potential conflict of interest.

## Publisher’s note

All claims expressed in this article are solely those of the authors and do not necessarily represent those of their affiliated organizations, or those of the publisher, the editors and the reviewers. Any product that may be evaluated in this article, or claim that may be made by its manufacturer, is not guaranteed or endorsed by the publisher.

## Footnotes

^1^https://wwwn.cdc.gov/nchs/nhanes/tutorials/default.aspx
